# Incidence of venous thromboembolism with intraoperative tranexamic acid use in breast surgery patients on estrogen-containing medications: a case series

**DOI:** 10.1016/j.jpra.2026.09.021

**Published:** 2026-09-23

**Authors:** Quentin K. Tsang, Isabella F. Churchill, Precious Adekoya, Ammara Ghumman

**Affiliations:** a Michael G. DeGroote School of Medicine, McMaster University, Hamilton, Ontario, Canada; b Division of Plastic and Reconstructive Surgery, McMaster University, Hamilton, Ontario, Canada; c School of Medicine, Queen’s University, Kingston, Ontario, Canada; d Division of Plastic, Reconstructive and Aesthetic Surgery, University of Toronto, Toronto, Ontario, Canada

**Keywords:** Breast surgery, Venous thromboembolism, Tranexamic acid, Estrogens, Contraceptives, Oral, Combined, Case series

## Abstract

Consideration of perioperative bleeding and venous thromboembolism (VTE) are important thrombotic complications for surgeons. Tranexamic acid (TXA) is an antifibrinolytic agent that is often used perioperatively to control bleeding. Product monographs caution its use in patients using estrogen-containing medications due to theoretical increased risk of VTE. However, no studies have explored this relationship in breast surgery. This retrospective case series aimed to determine the incidence of VTE with intraoperative TXA use in breast surgery patients using estrogen-containing medication at our institution. Demographics, estrogen medications, surgical characteristics and incidence of VTE were recorded and summarized narratively. Between 2018–2025, 188 breast surgeries with intraoperative TXA administration were performed. Twelve patients used to estrogen-contain medications at the time of surgery. Eight patients used an estrogen-containing combined hormonal contraceptive. Four patients used estrogen hormone therapy. TXA was administered intravenously (N = 7) or topical irrigation (N = 5). No patients had incidence of VTE. This is the first study to describe the relationship of TXA and estrogen-containing medications in breast surgery patients. These results provide preliminary clinical data regarding a patient population for which TXA use is cautioned against. Future prospective studies are warranted to better define the risk-benefit profile of TXA in this population.

## Introduction

Prevention of bleeding is an important consideration for plastic surgeons to prevent complications such as hematoma and infection. Tranexamic acid (TXA) is a lysine-derivative antifibrinolytic that inhibits the conversion of plasminogen to plasmin, thereby preventing fibrin clot degradation, resulting in hemostasis and reduced blood loss.^1^ Its use has shown clinical benefit in surgical specialties, including plastic surgery.^1^^,^^2^ However, there have been theoretical concerns of venous thromboembolism (VTE) due to its antifibrinolytic properties that maintain clot formation.^1^^,^^3^ TXA use in patients with moderate to high coagulable states, such as exogenous estrogen use, is not recommended in plastic surgery patients due to limited evidence regarding the incidence of VTE with its use.^2^^,^^4^ Additionally, product monographs for TXA caution against its use in patients receiving exogenous estrogen.^3^ To date, there are no studies that have evaluated whether TXA use increases VTE in breast surgery patients using estrogen-containing medications. Given this gap in the literature and ongoing clinical caution, we aimed to describe the incidence of VTE in patients using combined hormonal contraceptives (CHCs) or estrogen hormonal therapy undergoing breast surgery with perioperative administration of tranexamic acid.

## Methods

This retrospective case series was approved by our local research ethics board. Written consent was waived. Charts of patients from a single institution who underwent breast surgery and perioperative TXA administration between January 1, 2018 and January 31, 2025 were identified using billing codes. Inclusion criteria were: (1) women ≥18 years of age; (2) received perioperative TXA; (3) using an estrogen-containing medication at the time of surgery. Patients with history of VTE thrombophilia were excluded. Patient data, including any incidence of VTE or surgical complications, until the last postsurgical follow-up were collected from electronic medical records and extracted by two independent reviewers. Incidence of VTE detection was based on clinical symptoms and confirmed with ultrasound as per institutional guidelines. Caprini score was calculated using data available through electronic record to determine the perioperative risk of VTE. Data was analyzed descriptively for individual patients.

## Results

Over 7 years, 188 breast surgery patients received perioperative TXA. Twelve patients were on an estrogen-containing medication at the time of breast surgery (CHCs containing estrogen: N = 8; estrogen hormone therapy: N = 4). No patients discontinued their estrogen-containing medication perioperatively. One patient (8.3%) was prescribed oral TXA prior to breast surgery, which she continued to use postoperatively without complications (Table 1). Ten patients underwent bilateral breast reduction mammoplasty, one patient had a left partial mastectomy with subsequent drainage of breast abscess, and one patient had bilateral breast implant removal, exchange and partial capsulectomy (Table 2). All 12 patients received intraoperative TXA (7 intravenous TXA 1000 mg; 5 patients' topical irrigation of TXA diluted in sterile saline). No patients received VTE prophylaxis because all procedures were day surgeries. Ten patients had a moderate risk (0.7%) of VTE while two patients had a high risk (1.8%) of VTE as calculated by the Caprini score. Despite moderate to high risk of VTE in our cohort, there were no reported incidences of VTE during a median follow up of 63 days.

**Table 1:** Characteristics of estrogen-containing medications used by participants.

Participant	Generic name	Purpose	Type	Route & Frequency	Estrogen dose	Progestin dose
*1*	Desogestrel/ethinyl estradiol	Birth control	Combined estrogen-progestin	PO daily	30 mcg	150 mcg
*2*	Levonorgestrel/ethinyl estradiol	Birth control	Combined estrogen-progestin	PO daily	20 mcg	100 mcg
*3*	Levonorgestrel/ethinyl estradiol	Birth control	Combined estrogen-progestin	PO daily	20 mcg	100 mcg
*4*	Estradiol	Hormone replacement therapy	Estrogen	Transdermal, daily	37.5 mcg	
*5*	Levonorgestrel/ethinyl estradiol	Birth control	Combined estrogen-progestin	PO daily	20 mcg	100 mcg
*6*	Desogestrel/ethinyl estradiol	Birth control	Combined estrogen-progestin	PO daily	30 mcg	150 mcg
*7*	Estradiol	Hormone replacement therapy	Estrogen	Transdermal, daily	0.75 mg	
*8*	Estradiol	Hormone replacement therapy	Estrogen	Vaginal, 2x weekly	10 mcg	
*9*	Levonorgestrel/ethinyl estradiol	Birth control	Combined estrogen-progestin	PO daily	30 mcg (active pill) 10 mcg (inactive pill)	150 mcg
*10*	Levonorgestrel/ethinyl estradiol	Birth control	Combined estrogen-progestin	PO daily	20 mcg	100 mcg
*11*	Estradiol	Hormone replacement therapy	Estrogen	PO daily	2 mg	
*12*	Norethindrone acetate/ethinyl estradiol	Birth control	Combined estrogen-progestin	PO daily	10 mcg	1 mg

**Table 2:** Surgical characteristics including surgery type, operative time, TXA use, Caprini score and incidence of VTE. Caprini score was calculated using data available through electronic medical records.

Participant	Type of surgery	Operative time	TXA dosage & route of administration	Smoking (Y/N)	Caprini score	VTE (Y/N)
*1*	Bilateral breast reduction mammoplasty	145	IV 1000 mg intra-op	N	4	N
*2*	Left partial mastectomy, abscess drainage of left breast	77	IV 1000 mg intra-op	N	4	N
*3*	Bilateral breast reduction mammoplasty	169	Irrigation 500 mg intra-op of right breast	Y	4	N
*4*	Bilateral breast reduction, left and right chest wall liposuction	124	IV 1000 mg intra-op	N	5	N
*5*	Bilateral breast reduction mammoplasty	185	IV 1000 mg intra-op	N	4	N
*6*	Bilateral breast reduction, left and right chest wall liposuction	136	Irrigation 1000 mg intra-op for both breasts	N	5	N
*7*	Revision bilateral breast reduction, left side areola reset, right side no incision on areola with inferior wedge bilaterally	121	Irrigation 1000 mg intra-op for both breasts	N	4	N
*8*	Bilateral breast reduction, left and right chest wall liposuction	168	Irrigation 500 mg intra-op for both breasts	Y	5	N
*9*	Bilateral breast reduction, left and right chest wall liposuction	167	IV 1000 mg intra-op PO 1000 mg TID PRN	Y	4	N
*10*	Bilateral breast reduction	154	IV 1000 mg intra-op	N	4	N
*11*	Bilateral removal and replacement of breast implants, bilateral breast mound and scar revision, bilateral partial capsulectomy.	134	Irrigation 1000 mg intra-op for both breasts	N	4	N
*12*	Bilateral breast reduction	144	IV 1000 mg intra-op	N	4	N

## Discussion

The prevention of both VTE and unwanted surgical bleeding are critical considerations for surgeons to reduce morbidity and mortality. We are the first to investigate the incidence of VTE in breast surgery patients on CHCs or estrogen hormone therapy that received intraoperative TXA. Notably, no VTE events occurred in this moderate to high-risk population taking exogenous estrogen. While the median follow-up is adequate for detecting VTE events, the relatively short duration of follow up is a limitation of this study. Another limitation is the route of TXA administration differed among our cohort (i.e., intravenous vs. topical irrigation). However, previous literature suggests comparable safety and efficacy between administration routes.^5^

Estrogen-containing medications create a procoagulant state as estrogen increases the activity of factors II, VII, X and XII and fibrinogen, which all act to promote coagulation.^4^ Furthermore, estrogen decreases anti-coagulant factors such as antithrombin, protein C and protein S.^4^ However, pharmacologically, TXA acts as an antifibrinolytic to prevent the breakdown of fibrin clots, rather than directly activate or augment the coagulation cascade and does not further increase the hypercoagulable state caused by estrogen.^3^ The current contraindication of TXA with exogenous estrogen may be due to anecdotal evidence, exclusion of women using estrogen-containing medication from clinical trials and post-marketing reports. Thus, while there may be a theoretical increased risk of VTE in patients using estrogen-containing medications, this may not translate to clinical significance as they have different impacts on hemostasis.

## Conclusion

This case series provides preliminary evidence on the incidence of VTE among breast surgery patients on estrogen-containing medications receiving perioperative TXA. While no VTE events were observed in this specific cohort, these findings serve as a foundation for larger, prospective studies to further evaluate the safety profile of TXA in this high-risk patient population.

## Ethical approval

This study was approved by the Hamilton Integrated Research Ethics Board (Project #: 18768) in accordance with the Tri-council Policy Statement on Ethical Conduct on Research Involving Humans (TCPS 2) and Canadian Guidelines on Good Clinical Practice (GCP).

## Funding

none.

## CRediT authorship contribution statement

**Quentin K. Tsang:** Conceptualization, Data curation, Formal analysis, Investigation, Methodology, Project administration, Resources, Software, Validation, Visualization, Writing – original draft, Writing – review & editing. **Isabella F. Churchill:** Conceptualization, Data curation, Formal analysis, Investigation, Methodology, Project administration, Resources, Software, Validation, Visualization, Writing – original draft, Writing – review & editing. **Precious Adekoya:** Formal analysis, Visualization, Writing – original draft, Writing – review & editing. **Ammara Ghumman:** Conceptualization, Methodology, Project administration, Resources, Supervision, Validation, Writing – original draft, Writing – review & editing.

## Declaration of competing interest

QKT, PA, IFC and AG do not have any conflicts of interest to disclose.
